# Current Gene Expression Studies in Esophageal Carcinoma

**DOI:** 10.2174/138920209789503888

**Published:** 2009-12

**Authors:** Wei Guo, Yao-Guang Jiang

**Affiliations:** Department of Thoracic Surgery, Institute of Surgery Research, Daping Hospital, Third Military Medical University, Chongqing, P.R. China

**Keywords:** Esophageal cancer, DNA microarray, gene expression.

## Abstract

Esophageal carcinoma is one of the deadliest cancers with highly aggressive potency, ranking as the sixth most common cancer among males and ninth most common cancer among females globally. Due to metastasis and invasion of surrounding tissues in early stage, the 5-year overall survival rate (14%) of esophageal cancer remains poor, even in comparison with the dismal survival rates (4%) from the 1970s. Numerous genes and proteins with abnormal expression and function involve in the pathogenesis of esophageal cancer, but the concrete process remains unclear. Microarray technique has been applied to investigating esophageal cancer. Many gene expression studies have been undertaken to look at the specific patterns of gene transcript levels in esophageal cancer. Human tissues and cell lines were used in these geneprofiling studies and a very valuable and interesting set of data has resulted from various microarray experiments. These expression studies have provided increased understanding of the complex pathological mechanisms involved in esophageal cancer. The eventual goal of microarray is to discover new markers for therapy and to customize therapy based on an individual tumor genetic composition. This review summarized the current state of gene expression profile studies in esophageal cancer.

## INTRODUCTION

As one of the deadliest cancers with highly aggressive potency, esophageal cancer (EC) ranks as the sixth most common cancer among males and ninth most common cancer among females globally [1]. Worldwide, EC is the sixth leading cause of death from cancer [2]. EC is of two main types, each with distinct etiological and pathological characteristics. Esophageal squamous cell carcinoma (ESCC) is predominant type of ECs worldwide comprising more than 90% of all cases [3]. While ESCC is prevalent in the developing world, esophageal adenocarcinoma is commonly seen in the developed country, usually in association with Barrett's esophagus. Due to metastasis and invasion of surrounding tissues in early stage, the 5-year overall survival rate (14%) of EC remains poor, even in comparison with the dismal survival rates (4%) from the 1970s [4]. Numerous genes and proteins with abnormal expression and function involve in the pathogenesis of EC, but the concrete process remains unclear. We still have limited understanding of the pathophysiology of this disease and a lack of a diagnostic serum marker.

The term DNA microarray (also called DNA chip) refers to the systematic arrangement of biomolecular probes such as DNA molecules on a solid surface. Either cDNA microarrays or oligonucleotidebased chips may be used for gene expression analysis. Microarray technique was firstly used in and then was utilized in the field of gene expression since 1995 [5]. A high-capacity system was developed to monitor the expression of many genes in parallel. Microarrays prepared by high-speed robotic printing of complementary DNAs on glass were used for quantitative expression measurements of the corresponding genes. Because of the small format and high density of the arrays, hybridization volumes of 2 microliters could be used that enabled detection of rare transcripts in probe mixtures derived from 2 micrograms of total cellular messenger RNA. Differential expression measurements of 45 *Arabidopsis* genes were made by means of simultaneous, two-color fluorescence hybridization [5]. Microarray technique allows us to monitor the expression of thousands of genes simultaneously and has been used successfully to explore the gene expression of carcinoma and other diseases [6-9].

DNA microarray has been utilized in the studies of EC since 2001, several microarray studies were performed for investigating the gene expression profiling in EC tissue and cell lines [10-12]. Gene expression profiling studies promise to provide a more functional molecular understanding of this disease. In this review, we systematically examined the published results from microarray-based outcome studies in EC. Moreover, we presented associations between gene expression profiles and tumor metastasis, chemoradiotherapy resistance, immunotherapy and patient survival.

## GENE EXPRESSION PROFILES AND METASTASIS

Metastasis and invasion of surrounding organs are the major wrongdoers for the poor prognosis of EC. Up to date, the tumor, node, metastasis (TNM) staging system is still the primary method for determining the extent of the cancer and the prognosis of patients, and it often functions as a surrogate for survival. However, due to the existence of undetectable micrometastasis and low sensitivity of clinical imaging, this system does not always predict prognosis accurately. Therefore, finding and identifing of new molecular markers related to the prognosis of patients is a promissing method for achieve more accurate clinical outcome predictions and treatment options of EC.

Lymph node metastasis, including the number and location of lymph nodes involved, is one of the most important determinants in distinguishing early-stage and advanced-stage EC. A focus in EC molecular profiling is to compare gene expression profiles of tumors with lymph node metastasis and those without to find a signature that can predict lymph node status of a primary tumor. Since 2003, there were several studies focused on the potential specific biomarkers for predicting and detecting the lymph node metastasis in EC [13-16]. By the aid of cDNA microarray analysis, Kawamata *et al.* [13] compared the expression profiles of 9,206 genes in metastasizing human ESCC cell line T.Tn-AT1 to its parental non-metastasizing cell line. They identified 34 genes showed more than 3-fold differential expression in T.Tn-AT1 cells and confirmed the expression levels of 14 of these genes by means of RT-PCR. The encoded proteins of these genes associated with adhesion, migration, inflammation, proliferation and differentiation regulation. They hypothesed these genes might regulate the metastasis of ESCC, and could be predictive markers for lymph node metastasis.

After investigating the gene expression profile in tumor tissue of 28 cases of ESCC by cDNA microarray, Kan and his colleagues [14] utilized analyzing artificial neural network (ANN) model to predict occurrence of lymph node metastasis. They found that it was difficult to extract useful information for the prediction of lymph node metastasis by clustering analysis. But systematic analysis combining Significance Analysis of Microarrays with ANN was very useful for the prediction of lymph node metastasis in ECs. This finding provided an useful method for the detecting the metastasis of lymph node in ECs.

Uchikado *et al.* [15] used oligonucleotide DNA chips that included a total of 17, 086 probes to investigate the genes related to lymph node metastasis in ESCC. The non-cancerous paired tissues were chosen for control and the pathological examination of lymph node dissection was also reviewed. This resulted in the identification of 43 genes that were overexpressed and 138 genes were down-regulated in ESCC compared to non-cancerous paired tissues. These altering expressing genes, involved in cell-cycle and cell adhesion regulation, apoptosis, and cell differentiation related. The expression of 5 overexpressed genes and one suppressed expression gene were confirmed by real-time semi-quantitative reverse transcriptional polymerase chain reaction (RT-PCR) method not only in study cases but also in additional 21 cases. Their result of real-time semi-quantitative RT-PCR was in accordance with the microarray data.

Another study performed to find the relationship between gene expression profile and metastasis was presented by Wong *et al.* [17]. Using 15 adjacent normal/tumor-matched ESCC tissues as the specimens, they identified 40 up-regulated and 95 down-regulated genes and verificated the microarray measurement by quantitative real-time reverse transcription PCR. After comparing the data with protein-protein interaction databases, 18 genes were presumed as the minimal discriminators for distinguishing ESCC tumors from normal specimens. Ten of them were associated with tumor metastasis.

These studies not only reveal how novel insights can be obtained from gene expression profiling, but also highlight a group of highly interacting genes associated with metastasis in ESCC. We were able to extract some genes related to nodal metastasis in ESCC by microarray technique. However, further examination is necessary in other genes as well as the interaction between the cancer cells and the stromal tissues.

## GENE EXPRESSION PROFILES AND CHEMOTHERAPY SENSITIVITY

Surgery remains the standard primary treatment and the best palliation for patients with EC. Traditional management of patients with localized EC has been dominated by surgical resection. However, the survival is still poor and many patients develop metastatic disease or locoregional recurrence after surgery [18]. As an alternative to resection for locoregional treatment of EC, there is some evidence to support combined chemoradiotherapy over radiotherapy alone [19].

Both squamous-cell carcinoma and adenocarcinoma of the esophagus are responsive to chemotherapeutics, thus making chemotherapy as an available treat of EC. According to previous reports of Enzinger and his colleagues, shrinkage of the EC by at least 50 percent may occur in 15 to 30 percent of patients who are treated with fluorouracil, taxane (paclitaxel or docetaxel), or irinotecan [20]. More studies proved that similar responses was observed in 35 to 55 percent of patients who received cisplatin (CDDP) in combination with these chemotherapeutics [21-26]. However, for many patients with EC, chemotherapy is plagued by the drug resistance, which can be both intrinsic and acquired. Therefore, it is of undoubted importance to explore the mechanism of the resistance to chemotherapeutics in EC.

As one of the most widely used platinum-containing anticancer drugs of chemotherapy for EC, CDDP is believed to induce tumor cell death as a result of the formation of CDDP-DNA adducts, which inhibit DNA replication and transcription [27]. Up to date, the presence of intrinsic or acquired resistance to CDDP in esopgageal cancer cells remains a major obstacle to successful chemotherapy. Therefore, gaining insight of the precise mechanisms of CDDP resistance and reversing it would provide the new strategies for cancer therapy. It is known that the mechanisms of resistance to CDDP are multifactorial, many genes or gene products have been reported to be responsible for CDDP resistance [28]. And it was well known that a number of cellular adaptations, including reduced uptake, inactivation by glutathione and other anti-oxidants, and increased levels of DNA repair or DNA tolerance, could cause the drug-resistance, which may contribute to the poor survival of EC.

In 2001, Kihara *et al.* [29] used cDNA chips that included a total of 9,216 probes to investigate the expression profiles of 20 EC tissues from patients who were treated with the same adjuvant chemotherapy after removal of tumor by operation, and attempted to find genes associated with the duration of survival after surgery. By comparing expression profiles of those cancer tissues, they identified 52 genes that were likely to be correlated with prognosis and possibly with resistance to anticancer drugs. These genes including H.sapiens H4/j gene, c-erbB2 and epidermal growth factor receptor.

Toshimitsu and his colleagues [30] utilized cDNA microarray technology to explore the potential genes involved in acquired CDDP resistance of ESCC. They choose a CDDP-resistant cell line (YES-2/CDDP) as the objects, which show resistance to CDDP and decreasing CDDP-accumulation compared with the parental YES-2 ESCC cell line. They identified 44 genes with significantly different expression levels between YES-2/CDDP and YES-2 cells and found that 15 of these 44 genes encoded ribosome-related proteins, almost all of which were underexpressed in YES-2/CDDP cells. These results suggested that the ribosome-related genes might involve in the acquired resistance to CDDP in ESCC.

A complete pathological response to chemotherapy for EC is associated with favourable survival, therefore, identidication the drug-response predictor is of great importance to the chemotherapy for EC. However, prediction of therapeutic efficacy is hampered by the intricate mechanism of drug sensitivity. Fumoto *et al.* [31] used oligonucleotide DNA chips to identify novel potent marker genes related to sensitivity for CDDP and 5-Fu in ESCC cell lines. After investigating the gene expression profiles of 20 KYSE human ESCC cell lines and 18 tumor samples, they demonstrated that interferon induced transmembrane protein 1 (IFITM1) gene was possibly a key determinant of the CDDP sensitivity. And found that a set of the selected genes including IFITM1 maybe effective for predicting therapeutic responses to CDDP chemotherapy in EC. These results might provide a new method to predict the therapeutic efficacy of CDDP and 5-Fu in EC.

Our previous study [32] revealed that the upregulation of zinc ribbon domain-containing-1 (ZNRD1) gene could significantly enhance the tolerance of ESCC cell to CDDP. We investigated the gene ZNRD1-related expression profile by cDNA microarray analysis in ESCC cells and identified 16 genes with significantly different expression levels. Our results suggested that and overexpression of ZNRD1 could enhance the resistance to CDDP in human ESCC cells by upregulation of excision repair cross-complementing-1 (ERCC1) and B-cell lymphoma-2 (Bcl-2).

Recently, Takashima *et al.* [33] using an oligonucleotide microarray consisting of 34, 594 genes to identify the genes that are related to the sensitivity to CDDP in EC cells. The IC50 for CDDP was measured for 15 EC cell lines, then the microarray was utilized to explore the different gene expression level. They identified 37 genes were differentially expressed in the CDDP-resistant EC cell line. Their results might provide several candidate genes that may be associated with resistance to CDDP in EC cells, but the further functional studies is still necessary to confirm the possible effect of these genes in CDDP resistance.

## GENE EXPRESSION PROFILES AND RADIOTHERAPY RESISTANCE

The use of primary radiotherapy as an alternative to surgery was initially evaluated in patients with ESCC whose general medical condition made them poor operative candidates. The most important advantage of primary radiotherapy is the avoidance of perioperative morbidity and mortality [4]. However, the resistance to radiotherapy hampered the patients acquire more excellent therapeutic efficacy and contributed the poor prognosis of EC. Microarray technique provides a new method to gain insights into the molecular mechanisms underlying the effect of irradiation on EC.

In 2004, Bo *et al.* [34] used a cDNA microarray screening of more than 4,000 genes with known functions to identify genes involved in the early response to ionizing irradiation on ESCCs. According to their results, there were 27 genes overexpressed and 26 downregulated in ESCC cell lines after ionizing irradiation. The author utilized semiquantitative RT-PCR to partly identify the results of microarray. However, this observation can only suggest the relationship between these genes and the irradiation-reaction of EC cells. Further study is still needed to understanding of the molecular basis of radiotherapy and in developing strategies to augment its efficacy.

Fukuda and his colleagues [35] established radioresistant EC cell lines by applying fractionated irradiation, and then identified the differentially expressed genes between parent and radioresistant cells. Using cDNA microarray consisting of 21,168 genes, they identified 19 genes upregulated and 28 downregulated common to radioresistant EC cell sublines. Reverse transcription-polymerase chain reaction was utilized to confirm that genes selected by cDNA microarray were overexpressed in clinical specimens of radioresistant cases. The upregulated genes were associated with apoptosis and inflammatory response (BIRC2 and COX-2), DNA metabolism (CD73), and cell growth (PLAU). The function of downregulated genes included apoptosis (CASP6), cell adhesion (CDH1 and CDH3), transcription (MLL3), and cell cycle (CDK6). Importantly, their results identified the relationship between COX-2 and radioation resistance proved by previous studies.

Recently, Ogawa *et al.* [36] analyzed the global gene expression profiles in radiosensitive and radioresistant ESCC cell lines using a 34, 594-spot oligonucleotide microarray. Totally, gene expression profiles of 13 EC cell lines were investigated compared with a normal esophageal epithelial cell line. They found the TE-11 cell line was highly sensitive to radiation and identified 71 candidate genes that were differentially expressed in TE-11 by microarray analysis.

## GENE EXPRESSION PROFILES AND IMMUNOTHERAPY

Up to data, the treatment of unresectable and relapsed ECs still remains a challenge, especially in patients acquiring resistance to presently available chemotherapy or radiation therapy regimens. Therefore, development of a new effective therapeutic approach such as immunotherapy is needed to expand treatment modalities [37, 38]. By the aids of cDNA microarray analysis, screening of tumor-associated antigens, we can identified the antigens strongly and specifically expressed only in cancer cells and not in normal cell counterparts. And then choosing these specifical antigens as the molecular target for immunotherapy.

In a promising study performed at 2004 [39], Yoshitake and his colleagues found a novel candidate antigen, proliferation potential-related protein (PP-RP) by cDNA microarray analysis. And examined cytotoxicity against tumor cells *in vitro* and *in vivo* of cytotoxic T lymphocytes (CTLs) specific to PP-RP established from EC patients. According to their results, expression of PP-RP in EC cells was significantly higher than in normal cells, and the CTLs recognizing PP-RP killed tumor cells *in vitro* and also showed tumor rejection effects in nude mouse engrafted human EC cells. This finding also proved that cDNA microarray analysis is a useful method to identify ideal tumor-associated antigens.

## GENE EXPRESSION PROFILES AND PATIENT SURVIVAL

During the past decades, the improvement of surgical skill and chemotherapeutics made the patients with EC could acquire better therapeutic efficacy. However, the 5-year overall survival rate of EC remains poor. The metastasis, even occuring in early stage, is the major wrongdoer of this depressed situation. The now modified TNM staging system provides adequate prognostic information for patients with esophageal. However, application of this staging system results in the potential overtreatment or undertreatment of a significant number of patients, and it can only be applied after complete surgical resection rather than after a presurgical biopsy. Moreover, detecting the micrometastasis still remains a challenge for clinicians and pathologists. Therefore, identifing new molecular markers for prognosis could provide important supplement for current TNM staging system of EC. As a high-throughput research method, microarray could provide convenience for finding and identifing the molecular markers for estimating the patient survival. Recently developed microarray technology has permitted the development of multi-organ cancer classifiers [40, 41] identification of tumor subclasses [42-44], discovery of progression markers [45, 46] and prediction of disease outcome in many types of cancer [47-49]. Therefore, by the aid of microarray analysis, potential information might also be acquired for estimating the patient survival of EC.

Comparing to other malignancies, such as non-small cell lung cancer and breast cancer, there is still insufficient study about the relationship between altering gene expression profiles and patients’ survival in EC. And the well-designed prospective studies with large samples and factor analysis will help us to obtain novel insights in this field.

## CONCLUSIONS

Gene expression profiling has been applied in a wide range of EC outcome studies and has revealed hundreds of significantly altered expressed genes. Some of these genes have been further investigated and have provided increased understanding of the complex pathological mechanisms involved in EC. However, a common problem in most published studies is lacking evaluation of the value-added utility of expression profiling results in the context of known prognostic factors. And due to lack of reference databases for comprehensive gene expression for human cancers and no specific group of biomarkers for the diagnosis of specific tumors, accurate diagnoses of individual tumors by gene expression profile alone are not always possible. In spite of these limitations, DNA microarray could be best used for molecular classification of EC based on genetic and biological changes. And microarray technique will help us to obtain novel insights in the highly interacting genes associated with carcinogenesis of EC.
